# Low IgA Associated With Oropharyngeal Microbiota Changes and Lung Disease in Primary Antibody Deficiency

**DOI:** 10.3389/fimmu.2020.01245

**Published:** 2020-06-19

**Authors:** Roos-Marijn Berbers, Firdaus A. A. Mohamed Hoesein, Pauline M. Ellerbroek, Joris M. van Montfrans, Virgil A. S. H. Dalm, P. Martin van Hagen, Fernanda L. Paganelli, Marco C. Viveen, Malbert R. C. Rogers, Pim A. de Jong, Hae-Won Uh, Rob J. L. Willems, Helen L. Leavis

**Affiliations:** ^1^Department of Rheumatology and Clinical Immunology, University Medical Center Utrecht and Utrecht University, Utrecht, Netherlands; ^2^Department of Radiology, University Medical Center Utrecht and Utrecht University, Utrecht, Netherlands; ^3^Department of Internal Medicine and Infectious Diseases, University Medical Center Utrecht and Utrecht University, Utrecht, Netherlands; ^4^Department of Paediatric Immunology and Infectious Diseases, University Medical Center Utrecht and Utrecht University, Utrecht, Netherlands; ^5^Division of Clinical Immunology, Department of Internal Medicine, Erasmus University Medical Center Rotterdam, Rotterdam, Netherlands; ^6^Department of Immunology, Erasmus University Medical Center Rotterdam, Rotterdam, Netherlands; ^7^Academic Center for Rare Immunological Diseases (RIDC), Erasmus University Medical Center Rotterdam, Rotterdam, Netherlands; ^8^Department of Medical Microbiology, University Medical Center Utrecht and Utrecht University, Utrecht, Netherlands; ^9^Department of Biostatistics and Research Support, University Medical Center Utrecht and Utrecht University, Utrecht, Netherlands

**Keywords:** microbiota, immunoglobulin A, lung disease, CVID, XLA, oropharyngeal

## Abstract

Common Variable Immunodeficiency (CVID) and X-linked agammaglobulinemia (XLA) are primary antibody deficiencies characterized by hypogammaglobulinemia and recurrent infections, which can lead to structural airway disease (AD) and interstitial lung disease (ILD). We investigated associations between serum IgA, oropharyngeal microbiota composition and severity of lung disease in these patients. In this cross-sectional multicentre study we analyzed oropharyngeal microbiota composition of 86 CVID patients, 12 XLA patients and 49 healthy controls (HC) using next-generation sequencing of the 16S rRNA gene. qPCR was used to estimate bacterial load. IgA was measured in serum. High resolution CT scans were scored for severity of AD and ILD. Oropharyngeal bacterial load was increased in CVID patients with low IgA (*p* = 0.013) and XLA (*p* = 0.029) compared to HC. IgA status was associated with distinct beta (between-sample) diversity (*p* = 0.039), enrichment of (*Allo)prevotella*, and more severe radiographic lung disease (*p* = 0.003), independently of recent antibiotic use. AD scores were positively associated with *Prevotella, Alloprevotella*, and *Selenomonas*, and ILD scores with *Streptococcus* and negatively with *Rothia*. In clinically stable patients with CVID and XLA, radiographic lung disease was associated with IgA deficiency and expansion of distinct oropharyngeal bacterial taxa. Our findings highlight IgA as a potential driver of upper respiratory tract microbiota homeostasis.

## Introduction

The microbiota of the respiratory tract is increasingly recognized as an important driver of respiratory health (1), and has been associated with susceptibility to infection (2), hypersensitivity reactions such as asthma (3), and immune-mediated lung disease such as sarcoidosis (4). The quantity and composition of the lung microbiota is determined by host defense mechanisms and mucociliary clearance, but originates in the upper respiratory tract, from where it migrates to the lung via micro aspiration or directly via the mucus layer (5).

IgA is thought to be important for the regulation of the microbiota at mucosal surfaces (6), but the effects of clinical IgA deficiency on respiratory tract microbiota homeostasis in humans remain uninvestigated (7). Studying the microbiota of patients with primary antibody deficiency such as common variable immunodeficiency (CVID) and X-linked agammaglobulinemia (XLA) can provide insight into the role and importance of the humoral immune system in controlling the microbiota (8).

The antibody deficiency in CVID is defined as low IgG, and either low IgA or IgM, or both (9). As a result, some CVID patients have residual IgA production and others are completely IgA deficient, and studying differences between these two CVID subgroups can provide information on the consequences of IgA deficiency, including on the microbiota (10, 11). While CVID can develop later in life and its causes are thought to be multifactorial (9), XLA is a congenital disease that is the result of a mutation in Bruton's tyrosine kinase, resulting in an early B cell defect and complete humoral immunodeficiency from birth, including IgG, IgA and IgM (12).

Despite immunoglobulin G replacement therapy (IgGRT) which limits the recurrence of (respiratory) infections (13), 16–25% of CVID patients develop structural airway disease (AD) and 3–19% develop interstitial lung disease (ILD) (14–16), causing significant morbidity and mortality in these patients (14). XLA patients are also treated with IgGRT and remain prone to develop AD [in 32–47%, (17–19)], but generally do not develop ILD (20).

AD—which includes bronchiectasis—may seriously compromise pulmonary function and prevention is, therefore, important for a patients' prognosis (21). AD is thought to result from recurrent lower respiratory tract infections, but may also progress in the absence of evident clinical infections (22). ILD in CVID may present as granulomatous lung disease, lymphoid interstitial pneumonia, organizing pneumonia and lymphoproliferative disorders, summarized as granulomatous—lymphocytic interstitial lung disease (GLILD). Causes of ILD in CVID are poorly understood and there is currently no consensus on therapeutic strategies (23). Better understanding of the mechanisms that cause (GL) ILD can contribute to improving clinical care of these patients.

In the gut, low IgA in CVID has been associated with changes of the microbiota, including reduced alpha diversity and expansion of Bacilli and Gammaproteobacteria, which correlated with increased LPS levels in plasma, suggesting increased microbial (product) translocation (10). Whether similar changes occur in the (upper) respiratory tract microbiota in CVID has not been determined.

We hypothesize that IgA deficiency in CVID and XLA may lead to changes in the microbial composition of the respiratory tract, which could contribute to the development of structural lung disease. As a first step in investigating this hypothesis, we characterized the composition of the oropharyngeal microbiota of CVID and XLA patients and correlated this with serum IgA levels and severity of lung disease.

## Materials and Methods

More detailed materials and methods can be found in the online Supplementary Material.

### Study Population

Patients were age 7 or over, and diagnosed with CVID or XLA according to the European Society for Immunodeficiencies (ESID) criteria (24). Partners, friends and family members of patients were included as healthy controls (HC). All CVID and XLA patients received immunoglobulin substitution therapy at time of sampling, with target IgG trough levels of >8.0 g/L. Clinical data was collected from the hospital electronic patient files.

### Serum Analysis

Serum was collected at time of microbiota sampling, and stored at −80°C until analysis. IgA was measured in serum using a PEG-enhanced immunoturbidimetric method (Atellica CH, Siemens). Very low IgA was defined as serum IgA <0.1 g/L, in order to be consistent with the first gut microbiota study in CVID by Jørgensen et.al. (10).

### Sample Collection and DNA Isolation

Oropharyngeal swabs (eSwab, Copan Innovation, Brescia, Italy) were collected by the treating physician or researcher and stored at −80°C the same day. DNA isolation was performed as described by Wyllie et al. (25).

### Bacterial Load qPCR

Bacterial load in the oropharyngeal swab samples was estimated using the BactQuant qPCR, as described by Liu et al. (26). Primers and probes were ordered from IDT DNA technologies. Forward primer: 5′- CCTACGGGDGGCWGCA-3', reverse primer: 5′- GGACTACHVGGGTMTCTAATC−3′, probe: (6-FAM/ZEN) 5′-CAGCAGCCGCGGTA-3′ (Iowa Black®FQ) (26). qPCR was performed using a StepOnePlus RT-PCR system (ThermoFisher).

### 16S rRNA Sequencing and Bioinformatics

The 469 basepair V3 and V4 hypervariable regions of the 16S rRNA gene were amplified and sequenced using the Illumina MiSeq instrument and Reagent Kit v3 (600-cycle) according to Fadrosh et al. (27). The resulting amplicon pool generated a total of 6.6 million reads. The QIIME2 microbial community analysis pipeline (version 2018.8) (28) was used with DADA2 (29) for sequence variant detection, and SILVA as 16S rRNA reference gene database (SILVA 132) (30). Sequencing data has been made available on the European Nucleotide Archive under project code PRJEB34684.

### Lung Disease Scores

High resolution CT (HRCT) volumetric scans were performed in routine diagnostic evaluation every 5 years according to local protocol for CVID and XLA patients, and was preferred as screening test over spirometry because of better prognostic performance in our dataset (31). Each scan was scored by a board-certified thoracic radiologist (F.M.H.) for AD and ILD in each lobe using a previously published scoring system (32, 33). AD was scored as extent and severity of bronchiectasis, airway wall thickening, mucus plugging, tree-in-bud and airtrapping. ILD was scored as extent and severity of opacities, ground glass, septa thickening and lung nodules. The obtained score was normalized by the maximum obtainable score. For one patient who had undergone lobectomy and one with atelectasis of a single lobe, the maximum obtainable score was adapted to exclude the missing lobe. In thirteen cases where expiratory scans were not available, airtrapping could not be evaluated and this element was removed from the score.

### Data Analysis and Statistical Methods

All analyses were performed using R 3.2.0 (34), and made publically available on Gitlab: https://gitlab.com/rberbers/cvid_mbiota_oral/. Continuous variables were compared using the Mann-Whitney rank test or a Student's *t*-test, depending on distribution of the data. Categorical variables were compared using a two-tailed Fisher's exact test. Averages were reported as mean **±** standard deviation (SD), or as median ± interquartile range (IQR). Inverse Simpson index was calculated using the package *vegan*. Principal Component Analysis (PCA) was performed using the *prcomp* function on centered log ratio (CLR) transformed data (35). PERMANOVA was used to detect global community differences in PCA using the package *vegan*. Differential abundance testing was performed using ANCOM.2 (36) with Benjamini-Hochberg correction for false discovery rate (FDR) using an alpha of 0.05 as a threshold for significance. All ANCOM analyses were corrected for age and gender.

In contrast to the dichotomous clinical ILD status of patients as reported in Table 1, continuous lung disease scores were used to determine correlation with microbiota. Linear regression was performed upon the CLR-transformed sequencing data as described above. using the function *lm*() and the following model: [lung score] ~ gender + age + [bacterium].

**Table 1:** Study population characteristics.

	**HC**	**CVID + IgA (IgA >0.1 g/L)**	**CVID -IgA (IgA < 0.1 g/L)**	**XLA**
Total N	49	50	36	12
Age: mean ± SD	42 ± 12	34 ± 19	38 ± 14	22 ± 15
Male % (N)	31% (15/49)	50% (25/50)	58% (21/36)	100% (12/12)
**MEDICATION USE DURING 3 MONTHS PRIOR TO SAMPLING: % (N)**				
Antibiotics	0% (0/49)	36% (18/50)	28% (10/36)	58% (7/12)
Immune suppressive therapy	0% (0/49)	12% (6/50)	14% (5/36)	8% (1/12)
**CLINICAL PHENOTYPE: % (N)**				
Any inflammatory complication	0% (0/49)	30% (15/50)	67% (24/36)	8% (1/12)
Autoimmune disease	0% (0/49)	18% (9/50)	39% (14/36)	8% (1/12)
GLILD (clinical diagnosis)	0% (0/49)	6% (3/50)	8% (3/36)	0% (0/12)
Granulomatous disease other	0% (0/49)	2% (1/50)	6% (2/36)	0% (0/12)
Enteritis	0% (0/49)	14% (7/50)	28% (10/36)	0% (0/12)
Malignancy	0% (0/49)	6% (3/50)	3% (1/36)	0% (0/12)
**IgA STATUS:**				
Serum IgA levels available (N)	80% (39/49)	100% (50/50)	100% (36/36)	100% 12/12
% serum IgA low (<0.1 g/L)	0% (0/39)	0% (0/50)	100% (36/36)	100% (12/12)
Serum IgA mean ± SD in g/L	2.06 ± 0.78	0.74 ± 0.57	0.07 ± 0.00	0.07 ± 0.00

*HC, healthy control; CVID, common variable immunodeficiency; SD, standard deviation; XLA, X-linked agammaglobulinemia. Serum IgA below the limit of detection was reported as 0.070 g/L*.

Bootstrapped confidence intervals were generated using the function *boot*() and 1,000 iterations. Benjamini Hochberg correction was used to correct for FDR.

## Results

### Study Population Characteristics and Serum IgA

Oropharyngeal swabs were collected from 86 CVID patients, 49 HC and 12 XLA patients (Table 1) in two academic hospitals in Rotterdam and Utrecht, the Netherlands. Household members of patients were included as healthy controls. CVID patients were categorized as having very low IgA (IgA < 0.1 g/L, CVID–IgA, *n* = 36) or residual to normal IgA serum levels (IgA > 0.1 g/L, CVID+IgA, *n* = 50). Serum IgA could be determined in 39/49 HC, and was normal (IgA > 0.7 g/L) for all HC tested. There were fewer males in the HC than in both CVID groups (proportions of male participants 31% HC, 50% CVID + IgA, 58% CVID-IgA), and the HC were older (mean age ± standard deviation SD, HC 42 ± 12, CVID+IgA 34 ± 19 and CVID-IgA 38 ± 15). Naturally, all XLA patients were male. There was more recent antibiotic use in XLA than in CVID (58 vs. 36% and 28% in the CVID groups). Immune suppressive therapy (*n* = 12) consisted of 5 patients on low-dose prednisone (5–12,5 mg), 3 patients on anti-TNF therapy, 1 on methotrexate, 1 on cyclosporin A, 1 patient on azathioprine and anti-TNF therapy, and 1 patient on hydroxychloroquine, prednisone and azathioprine.

Inflammatory complications in CVID, defined as autoimmune disease (AI cytopenia *n* = 8, alopecia *n* = 4, SLE/SLE-like disease *n* = 3, vitiligo *n* = 3, Sjögren Disease *n* = 2, arthritis (non-RA) *n* = 2, RA *n* = 1, AI gastritis *n* = 1, hepatitis *n* = 1, myositis *n* = 1), GLILD, other granulomatous disease (clinical and radiographic suspicion of cerebral granulomas *n* = 2, splenic granuloma *n* = 1), enteritis and/or malignancy (NHL *n* = 1, CLL *n* = 1, high-grade B cell lymphoma *n* = 1, pancreas adenocarcinoma *n* = 1, thyroid carcinoma *n* = 1) (Table 1) were more prevalent in CVID-IgA (67%) than CVID+IgA (30%) in this cohort (*p* = 0.001). Some patients suffered from more than 1 complication. Of these complications, autoimmune disease was the most common, with 39% of CVID-IgA and 18% of CVID + IgA suffering from autoimmune disease (*p* = 0.047). In addition, one patient with XLA suffered from juvenile idiopathic arthritis. Serum IgA levels were lower in CVID patients with (*n* = 23) than without (*n* = 63) autoimmune disease (mean ± SD 0.21 ± 0.24 g/L and 0.55 ± 0.60 g/L, respectively, *p* = 0.010, data not shown). There were no significant differences between CVID-IgA and CVID+IgA in the prevalence of GLILD (*p* = 0.691), other granulomatous disease (*p* = 0.569), enteritis (*p* = 0.169), and malignancy (*p* = 0.637) in this cohort.

### Increased Oropharyngeal Bacterial Load in CVID and XLA

**Figure 1:**
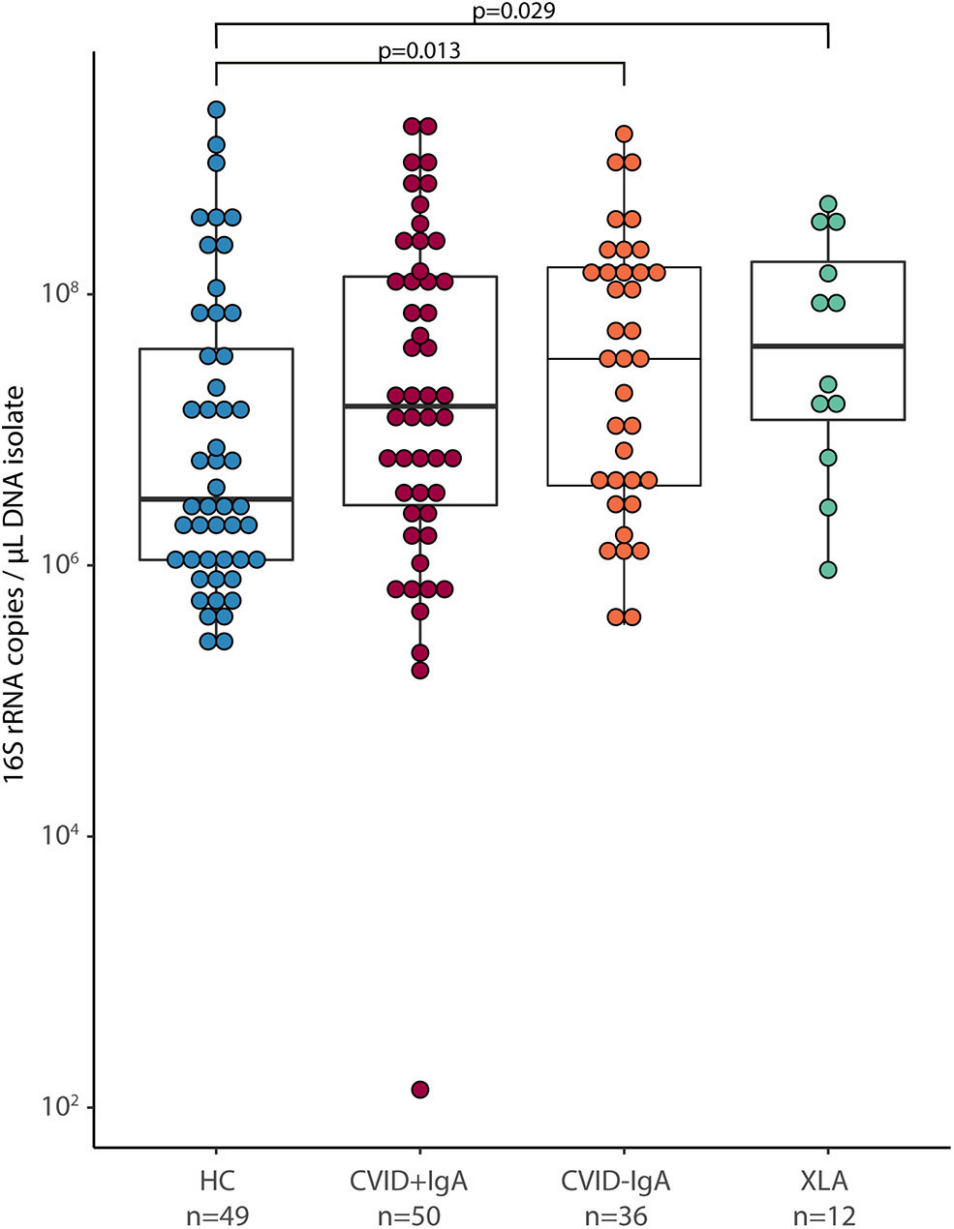
Bacterial load in oropharyngeal swab samples as determined by qPCR for copies of the 16S rRNA gene in DNA isolates from oropharyngeal swabs in healthy controls (HC, *n* = 49), CVID + IgA (*n* = 50), CVID –IgA (*n* = 36), and X-linked agammaglobulinemia (XLA, *n* = 12). The horizontal line inside the box represents the median. The whiskers represent the lowest and highest values within 1.5 × interquartile range. Statistical test: Mann-Whitney U test.

Bacterial load for each oropharyngeal swab sample was estimated by qPCR with 16S rRNA gene-based primers (Figure 1). There was a clear association between the median bacterial loads in oropharyngeal swabs and serum IgA deficiency: median bacterial load increased gradually as patients were more profoundly IgA impaired (median±interquartile range IQR: HC 3.1 × 10^6^ ± 38.4 × 10^6^, CVID+IgA 15.0 × 10^6^ ± 132.2 × 10^6^, CVID-IgA 33.4 × 10^6^ ± 154.3 × 10^6^, XLA 50.5 × 10^6^ ± 172.2 × 10^6^ copies of the 16S rRNA gene). Both CVID-IgA and XLA patients had significantly increased bacterial loads compared to HC (*p* = 0.013 and *p* = 0.029, respectively). The comparison of CVID+IgA vs. HC did not reach significance. As bacterial loads may be influenced by antibiotic use, we repeated analyses with patients who did not use antibiotics 3 months prior to sampling (Supplementary Figure 2), which yielded a broadly similar pattern.

### Alpha Diversity and Community Structure Differ in Absence of IgA

**Figure 2:**
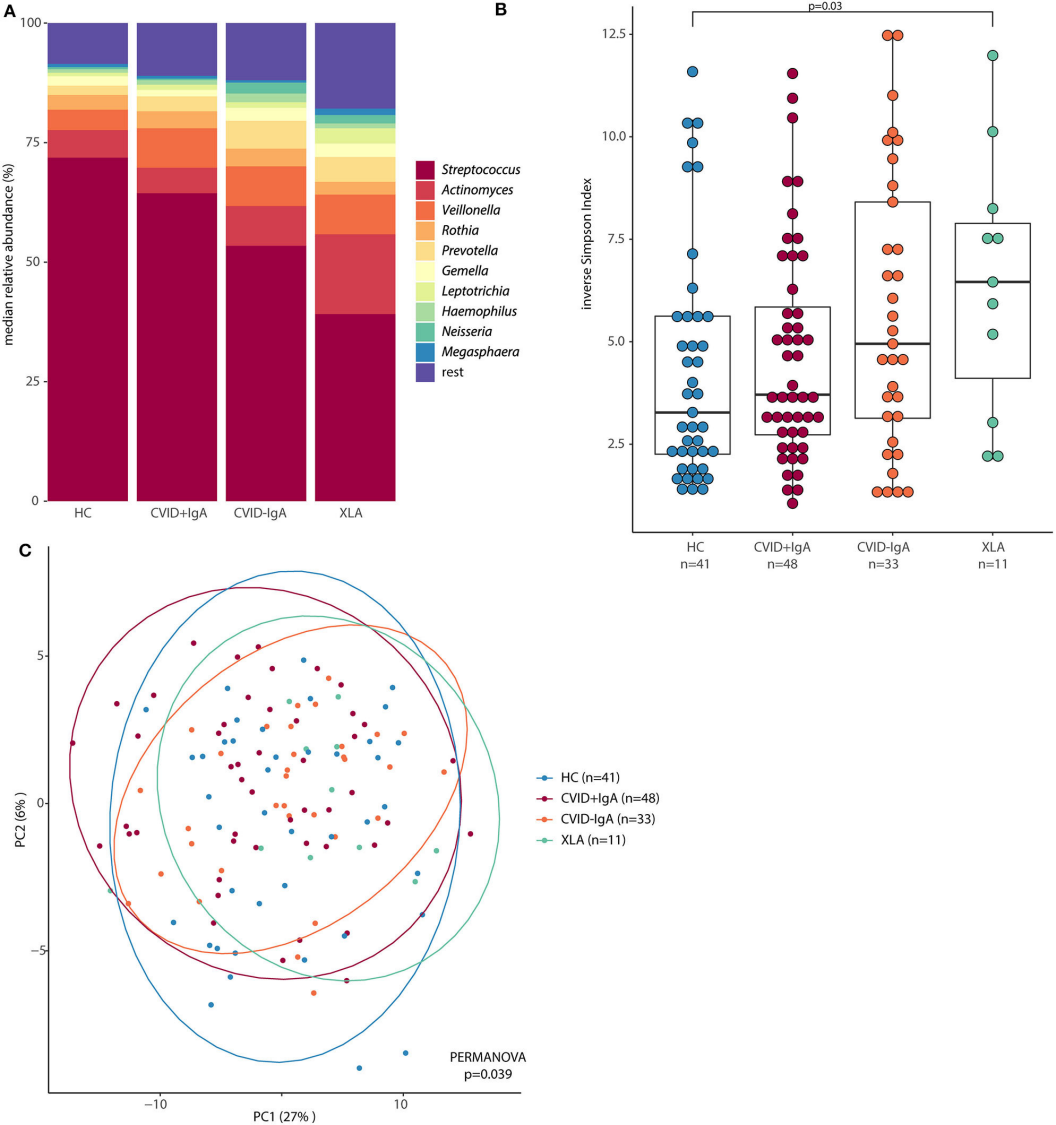
**(A)** Median relative abundance of top 10 most abundant genera determined by 16S rRNA sequencing of healthy controls (HC, *n* = 41), CVID +IgA (*n* = 48), CVID -IgA (*n* = 33), and XLA (*n* = 11). **(B)** Alpha diversity of the same samples described in **(A)** as measured by inverse Simpson's index on 16S rRNA sequencing data. The horizontal line inside the box represents the median. The whiskers represent the lowest and highest values within 1.5 × interquartile range. Statistical test: Mann-Whitney U test. **(C)** Principal component analysis of centered log ratio (CLR)-transformed family level 16S rRNA sequencing data of the same samples described in **(A)**. Ellipses indicate 95% confidence intervals. PERMANOVA using Euclidean distance on CLR data.

After 16S rRNA amplicon sequencing, 14 samples had insufficient sequencing coverage (<8,000 reads per sample) and were excluded from data analysis, leaving 41 HC, 81 CVID (48 CVID+IgA, 33 CVID–IgA) and 11 XLA samples (for an overview, please see Figure 1 and Table 1 of the Supplementary Materials). The overall top 10 most abundant bacterial genera were *Streptococcus, Actinomyces, Veillonella, Rothia, Prevotella, Gemella, Leptotrichia, Haemophilus, Neisseria*, and *Megasphaera* (Figure 2A).

Alpha diversity, a measure for the richness and evenness of a sample (expressed here as inverse Simpson index, Figure 2B), followed the same pattern as for bacterial loads; the lower the IgA, the higher the inverse Simpson index (median ± IQR: HC 3.28 ± 3.37, CVID+IgA 3.71 ± 3.12, CVID-IgA 4.95 ± 5.28, XLA 6.46 ± 3.78). These comparisons reached significance for XLA vs. HC (*p* = 0.03), unlike for CVID+IgA and CVID-IgA vs. HC. Similar results were observed in patients who were not using antibiotics at least 3 months prior to sampling (Supplementary Figure 3A).

Also the overall community structure (beta diversity) differed when grouping patients by IgA status (Figure 2C; *p* = 0.039). After excluding patients with recent antibiotic use (Supplementary Figure 3B), overall community structure remained significantly different between the groups (*p* = 0.039). Other comparisons did not reach significance.

### Expansion of Prevotellaceae Bacteria Associated With IgA Deficiency

**Figure 3:**
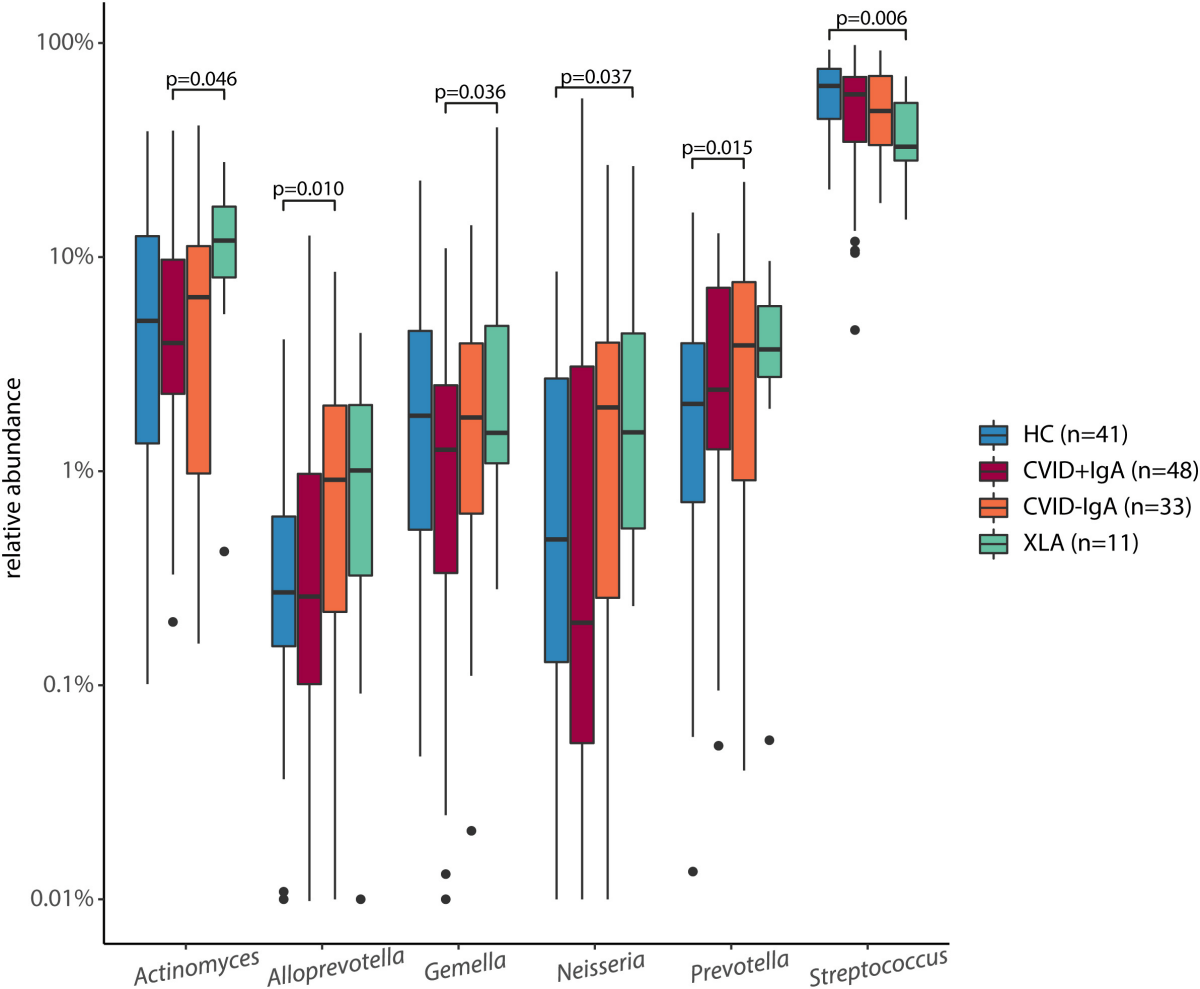
Differentially abundant taxa in 16S rRNA sequencing of healthy controls (HC, *n* = 41), CVID +IgA (*n* = 48), CVID -IgA (*n* = 33), and XLA (*n* = 11). Statistics: ANCOM corrected for age and gender and Benjamini-Hochberg correction for false discovery rate. The horizontal line inside the box represents the median. The whiskers represent the lowest and highest values within 1.5 × interquartile range. Y-axis shows relative abundance on log10 scale.

In order to further examine the differences in oropharyngeal microbiota in these patients, we next determined which bacterial genera were differentially abundant (Figure 3). Compared to HC, CVID-IgA patients had a higher relative abundance of two genera belonging to the Prevotellaceae family; *Prevotella* and *Alloprevotella* (*p* = 0.015 and *p* = 0.010, respectively). The same pattern of higher relative abundance of Prevotellaceae bacteria in more profound IgA deficiency was observed in patients without recent antibiotic use (Supplementary Figure 4). In XLA, relative abundance of *Prevotella* and *Alloprevotella* was similar to CVID-IgA but was not significant compared to HC, probably due to smaller sample size of the XLA group. Comparisons of Prevotellaceae among the other cohorts did not reach significance.

Other bacteria that were consistently differentially expressed in both the full cohort and the subgroups without recent antibiotic use were *Streptococcus* (decreased in XLA vs. HC, *p* = 0.006), and *Actinomyces* (increased in XLA vs. CVID+IgA, *p* = 0.046). Comparisons among the other cohorts did not reach significance.

### Radiographic Lung Disease Correlated With IgA Status, *Prevotella, Alloprevotella, Selenomonas*, and *Streptococcus*

**Figure 4:**
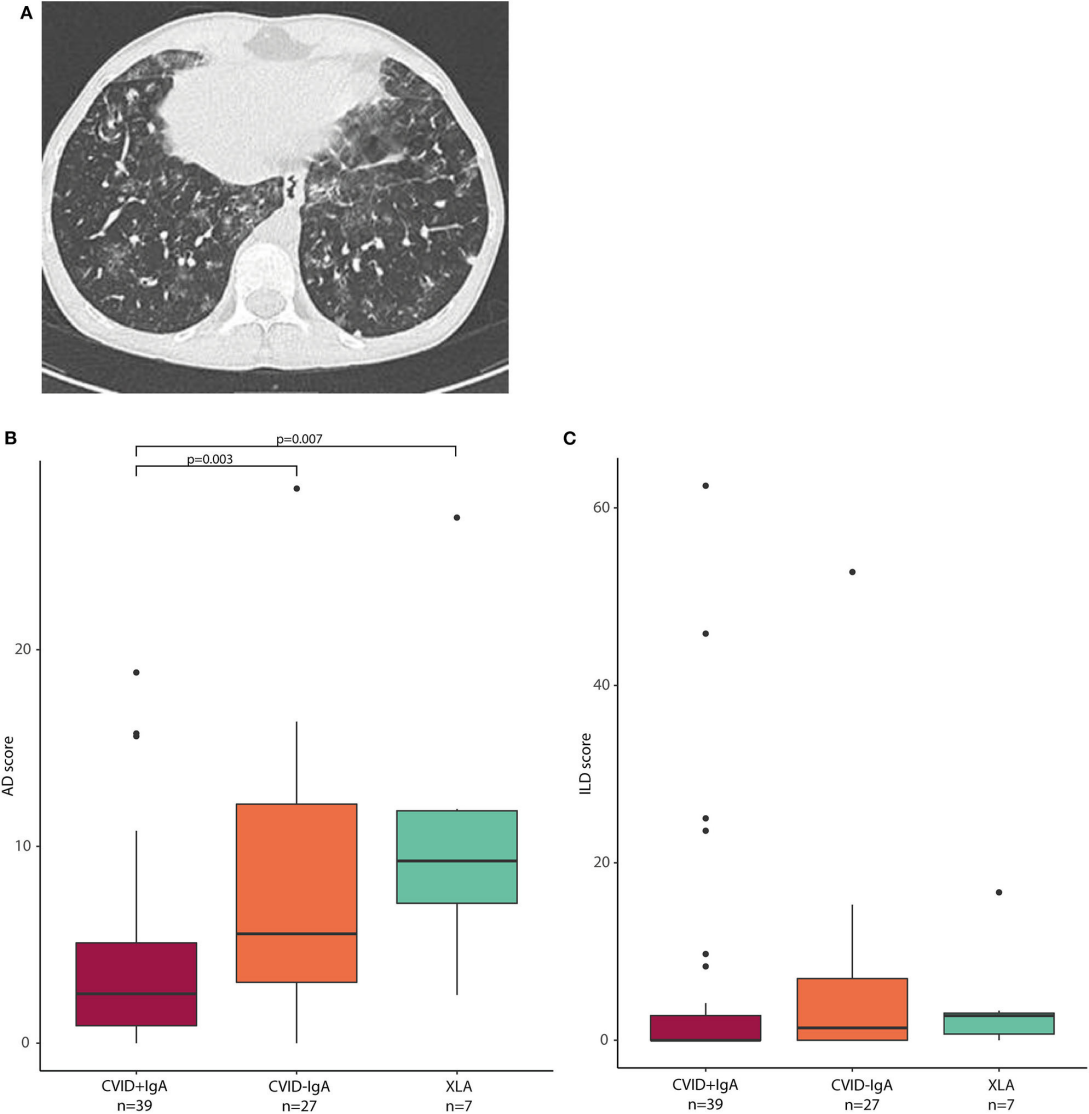
**(A)** Axial CT image: This 15-year old male patient with CVID had a total AD score of 13, and an ILD score of 48. Opacities, ground glass, septa thickening, and lung nodules were observed, especially in the lower lobes. **(B)** airway disease (AD) scores in CVID +IgA (*n* = 39), CVID -IgA (*n* = 27), and XLA (*n* = 7). **(C)** interstitial lung disease (ILD) scores in the same samples as in **(B)**. The horizontal line inside the box represents the median. The whiskers represent the lowest and highest values within 1.5 × interquartile range. Statistics: Mann-Whitney U test.

Next, we investigated whether the observed oropharyngeal microbiota differences in IgA deficient patients were associated with radiographic lung disease. From the group of patients in whom microbial community composition of oropharyngeal swabs was analyzed, high resolution chest CT scans were available for a total of 73 patients (39 CVID+IgA, 27 CVID-IgA and 7 XLA). Scans were performed for routine clinical follow-up, and mean time between scan and oropharyngeal swab was 1 year and 56 days (±1 year). For a representative scan, please see Figure 4A. Mean AD score in this population was 6.1 ± 6.0 with 27% of patients scoring below 2 and 23% scoring 10 or higher. Mean ILD score was 5.2 ± 11.4 with 64% scoring below 2 and 14% scoring 10 or higher. AD and ILD scores are used in this.

AD scores were higher in CVID patients with low IgA than in CVID+IgA (Figure 4B; CVID–IgA and XLA vs. CVID+IgA *p* = 0.003 and *p* = 0.007, respectively). No significant differences were observed for ILD score vs. IgA status (Figure 4C). Relationships between AD or ILD scores and bacterial load or alpha diversity were not detected here (Supplementary Figures 5A–C).

In linear regression corrected for age and gender including all CVID patients for whom scans were available (*n* = 66, see Table 2), *Prevotella* (*p* = 0.027, beta 95%CI 0.176, 1.654)*, Alloprevotella* (*p* = 0.029, beta 95%CI 0.004, 1.262)*, Selenomonas* (*p* = 0.023, beta 95%CI 0.166, 1.168) and *Megasphaera* (*p* = 0.032, beta 95%CI 0.142, 1.031) positively correlated with AD score, while *Fusobacterium* (*p* = 0.020, beta 95%CI −1.252, −0.048) and *Johnsonella* (*p* = 0.035, beta 95%CI −2.472, −0.949) correlated negatively (Table 2). *Streptococcus* (*p* = 0.001, beta 95%CI 0.257, 4.604) positively correlated with ILD scores, and *Rothia* was negatively associated with ILD score (*p* = 0.044, beta 95%CI −4.465, −0.434). None of the reported bacteria were significant after correction for false discovery rate, but all were stably detected after bootstrapping of samples to exclude effects due to outliers in the data.

**Table 2:** Linear regression with airway disease (AD) or interstitial lung disease (ILD) scores as dependent variable, and species level microbial sequencing data as independent variables, using data from 73 patients (39 CVID +IgA, 27 CVID –IgA, and 7 XLA).

**AD score**	**Beta**	***p*-value**	**95%CI (Beta)**	***R*^**2**^**
Age	0.057	0.189	−0.005, 0.146	
Gender	−0.062	0.966	−2.879, 2.266	
*Prevotella*	0.769	0.027	0.176, 1.654	0.099
*Alloprevotella*	0.682	0.029	0.004, 1.262	0.097
*Selenomonas*	0.650	0.023	0.166, 1.168	0.103
*Megasphaera*	0.551	0.032	0.142, 1.031	0.095
*Fusobacterium*	−0.704	0.020	−1.252, −0.048	0.107
*Johnsonella*	−1.577	0.035	−2.472, −0.949	0.093
**ILD score**	**Beta**	***p-*****value**	**95%CI (Beta)**	***R***^**2**^
Age	0.054	0.562	−0.142, 0.153	
Gender	2.180	0.483	−3.581, 9.215	
*Streptococcus*	2.173	0.001	0.257, 4.604	0.176
*Rothia*	−1.856	0.044	−4.465, −0.434	0.082

*Age and gender were included as covariates. Beta coefficient (beta) was bootstrapped to generate 95% confidence intervals (95%CI). Only taxa with p < 0.05 and 95% confidence intervals that do not span zero are reported*.

## Discussion

In this study we determined bacterial load and community composition of the oropharyngeal microbiota in CVID and XLA patients. We observed that bacterial load, alpha diversity and relative abundance of bacteria from the Prevotellaceae family were consistently increased in patients with more profound IgA deficiency, specifically CVID-IgA and XLA. Moreover, IgA deficiency and expansion of Prevotellaceae bacteria were associated with lung disease in these patients.

While IgA deficiency is one of the diagnostic criteria for CVID, some patients have residual IgA production, and others are almost completely IgA deficient. In XLA, no immunoglobulins are produced at all from birth. Despite IgGRT titrated to protect patients clinically against infection, oropharyngeal bacterial load was increased in CVID and XLA, and we observed a trend of increasing bacterial loads in patients with more profound IgA deficiency (HC < CVID+IgA < CVID–IgA < XLA). Control of bacterial load is most likely a multifactorial process, assumed to be affected by smoking habits, gastro-esophageal reflux [in Idiopathic Pulmonary Fibrosis (IPF)], air pollution, airway inflammation due to asthma/Chronic Obstructive Pulmonary Disease (COPD) and allergy, although literature is not conclusive (37–40). Nevertheless, our findings indicate that IgA may contribute to limiting the total amount of colonizing bacteria in the upper respiratory tract in these patients.

An additional consequence of increased bacterial load in CVID and XLA concerns interpretation of relative abundance data (41). As 16S rRNA gene based microbial profiling provides only compositional information, the increased total bacterial load in CVID and XLA means that any reported increase of relative abundance in these groups (such as for *Alloprevotella* and *Prevotella*) is expected to reflect a much greater increase in absolute bacterial numbers. The reverse may be the case for bacteria for which a relative decrease was reported (such as for instance in *Streptococcus*).

Concurrent with bacterial load, alpha diversity increased as patients were more profoundly IgA deficient, suggesting that IgA also limits the colonization of more different bacterial taxa. In gut microbiota high alpha diversity is generally associated with health, but this seems not to always apply to respiratory microbiota, as increased alpha diversity of the respiratory tract has been reported in other disease states such as asthma (42). Increased alpha diversity indicates a more complex alteration of the microbial composition rather than the outgrowth of a few pathobionts, which would result in decreased diversity as has been observed in infections (1) and acute exacerbations of non-CVID/XLA bronchiectasis (43).

Prevotellaceae genera were significantly expanded in CVID with low IgA levels and XLA. *Prevotella* are known mucus degraders (44) and have been associated with gut microbiome changes and immune-mediated disease (45). These bacteria are reportedly IgA coated in the gut (46) and were found to be increased in gut microbiota of patients with IgA deficiency and concurrent Th17 skewing (47). In the lung, *Prevotella*-derived outer membrane vesicles are thought to drive inflammation and fibrosis through the induction of Th17 responses upon TLR-2 activation (48). *Prevotella* may also drive inflammation indirectly by degrading mucins, as intact mucins can dampen innate immune responses by shielding bacterial ligands from TLRs (49). *Prevotella* has been associated with (exacerbations of) non-CVID bronchiectasis in other studies (50).

Relative abundance of *Prevotella* positively correlated with AD scores. This correlation was not significant after correction for false discovery rate, and additional studies will be needed to confirm the link between *Prevotella* (and the other detected bacteria) and airway disease. Increased relative abundance of *Streptococcus* was associated with higher ILD scores. Specific *Streptococcus* species have previously been linked with ILD progression in non-CVID patients in a prospective cohort study (51). Abundance of *Rothia* was negatively associated with ILD scores, suggesting a potential protective role. *Rothia* is described as part of the core microbiota of the oropharynx (52), and has also been found to be associated occurrence of pneumonia in elderly patients (2).

Strengths of this study are the integration between culture-independent microbiological community profiling, clinical immunology and pulmonary radiology. Limitations of this study include the cross-sectional nature, which does not allow for cause-effect distinctions (does IgA cause microbiota changes and do these cause lung disease, or are these bystander effects of a separate process?). There was a time delay between the oropharyngeal swabs, which were taken specifically for this study, and the chest CT scans, which are performed routinely every 5 years for clinical care in our clinics. Our group has previously shown that progression of radiographic AD and ILD scores over a 3–5-year follow-up period is very limited in CVID patients (33), and therefore the mean time between scan and swab of ~1 year in this study was deemed acceptable. We elected to use radiological evaluation in this study as previous work by our group has shown that the use of chest CT scans evaluated by a trained pulmonary radiologist is a superior predictor of early pulmonary abnormalities compared to pulmonary function testing in CVID (31). The number of XLA patients is limited compared to the CVID cohort, however, XLA is rare disease and it was therefore not feasible to include more patients in our studies. Also the number of patients with clinically significant ILD among CVID patients was limited (*n* = 6), which may limit external validity of ILD specific taxa from our cohort. A potential confounder in this study is use of antibiotics by CVID patients. Recent antibiotic use was recorded, and sensitivity analyses excluding all patients who had recently used antibiotics did not yield different insights as compared to analyses using the full cohort. However, an effect of long-term antibiotic use in this cohort cannot be excluded.

To conclude, we demonstrated that—despite IgGRT and independent of recent antibiotic use—patients with primary antibody deficiency carry an increased bacterial load in the upper respiratory tract, and have compositional changes of the oropharyngeal microbiota related to IgA deficiency. These compositional changes were associated with the radiographic presence of airway disease and interstitial lung disease. We speculate that IgA deficiency-induced changes in the microbiota composition of the respiratory tract may cause low-grade inflammation through increased microbial challenge, mucus degradation, and Th-17 skewing, resulting in inflammation-driven airway remodeling.

While the oropharyngeal microbiota has been found to partially overlap with pulmonary microbiota (53, 54), they are still distinct communities with important differences in bacterial load and composition (53, 54). Therefore, while our findings regarding oropharyngeal microbiota load and composition may reflect changes in the pulmonary microbiota indirectly through bacterial seeding of the lower respiratory tract from the oropharynx or similar consequences of IgA deficiency, further studies are required to show a direct, temporal and spatial relationship between IgA, microbiota and lung disease.

Direct interrogation of the lung microbiota, for instance through bronchial-alveolar lavage, can provide more information about local processes contributing to the development of lung disease. In addition, IgA-based therapeutic interventions in mouse models of antibody deficiency may help answer cause-effect questions, as well as provide first steps for better treatment in patients.

## Data Availability Statement

The datasets generated for this study can be found in the European Nucleotide Archive under project code PRJEB34684.

## Ethics Statement

Ethical approval for this study was received from the Medical Ethical Committee of the Erasmus Medical Center in Rotterdam, the Netherlands and the University Medical Center Utrecht in Utrecht, the Netherlands (METC: NL40331.078). Written informed consent was obtained from all patients (and in case of minors, their legal guardians) and controls according to the Declaration of Helsinki.

## Author Contributions

HL, R-MB, PE, JM, VD, and PH: study design and implementation. FM and PJ: radiological assessment. Wet lab work was performed by R-MB and MV. MR, FP, and H-WU: bioinformatics and statistical expertise. R-MB, HL, RW, and FP: data analysis and interpretation. All authors substantially contributed to the acquisition, analysis or interpretation of data for the manuscript and approved the final manuscript.

## Conflict of Interest

PH reports research grants and personal fees from Shire/Takeda and CSL Behring. VD reports research grants and personal fees from Shire/Takeda, Griffols, ACtelion, Novartis and CSL Behring. JM reports personal fees from Shire/Takeda. PJ reports research grants from Philips Healthcare. HL reports research grants and personal fees from Shire/Takeda. The remaining authors declare that the research was conducted in the absence of any commercial or financial relationships that could be construed as a potential conflict of interest.
