# Supplementary material for: Low IgA Associated With Oropharyngeal Microbiota Changes and Lung Disease in Primary Antibody Deficiency
Source: Front Immunol. 2020 Jun 19;11:1245. doi: 10.3389/fimmu.2020.01245 (PMC7318304; doi:10.3389/fimmu.2020.01245)
Supplement: Supplementary file 1 [file Data_Sheet_1.docx]

Supplementary Material

# Supplementary Methods

**1.1 Patient inclusion**

Patients were included at the outpatient clinics of the departments of clinical immunology, infectious diseases and paediatrics at the University Medical Center in Utrecht, the Netherlands, and the Erasmus Medical Center in Rotterdam, the Netherlands. Medication use in the three months prior to sampling was recorded. Clinical data was collected from the hospital electronic patient files. For laboratory measurements below the detection limit, the cut-off value was replaced with the limit of detection when computing mean serum levels. GLILD reported in the study characteristics was based on clinical assessment of the treating physician.

**1.2 DNA isolation**

DNA isolation was performed as described by Wyllie et al.^1^. Briefly, oral swab stored in 200uL of Amies medium was thawed and bead-beated twice in lysis buffer and phenol. The resulting DNA phase was purified using magnetic beads (LGC Genomics) and eluted into 100μL of DNA isolate. Negative controls and mock communities (ZymoBIOMICS microbial community (DNA) standard, Zymo research, USA) were used from the beginning of DNA isolation up to the data analysis stage.

## 1.3 Bacterial load qPCR

## Total bacterial load was determined using the BactQuant qPCR, as described by Liu et al.^2^, on a StepOnePlus RT-PCR system (ThermoFisher). Briefly, 2μL of undiluted DNA isolate for each sample was analysed with Taqman qPCR for 16S rRNA. Serial dilutions of a plasmid containing one *E.coli* 16S rRNA copy were used to calculate absolute 16S rRNA concentration in each sample. Primers and probes were ordered from IDT DNA technologies. TaqPath master mix (ThermoFisher) was used with standard cycling conditions on a StepOnePlus RT-PCR system (ThermoFisher). Forward primer: 5′- CCTACGGGDGGCWGCA-3’, reverse primer: 5′- GGACTACHVGGGTMTCTAATC -3′, probe: (6-FAM/ZEN) 5′-CAGCAGCCGCGGTA-3′ (Iowa Black®FQ).

**1.4 Bioinformatics**

The 469 basepair V3 and V4 hyper-variable regions of the 16S rRNA gene were amplified and sequenced using the Illumina MiSeq instrument and Reagent Kit v3 (600-cycle) according to Fadrosh et al. ^3^. The resulting amplicon pool generated a total of 6.6 million paired-end reads. These 2x300bp paired-end reads were pre-processed as follows. The first 12bp of each paired-end containing the index sequences, were extracted and afterwards concatenated to dual-index barcodes of 24bp specific for each read-pair and sample. Sequencing reads were de-multiplexed using the ‘qiime demux emp-paired’ command from the QIIME2 microbial community analysis pipeline (version 2018.8)^4^. The DADA2^5^ pipeline in QIIME2 using the command ‘qiime dada2 denoise-paired’ (with the options --p-trim-left-f 21 --p-trim-left-r 21 --p-trunc-len-f 275 --p-trunc-len-r 260) was then used for read quality filtering, removal of sequencing primers, paired-end reads merging, generating amplicon sequencing variants (ASVs) and the removal of chimeric sequences. After these steps, and removal of the control samples, a total of 3.2 million sequences were retained, with a mean amount of 19.3k per sample. The obtained ASVs (7351 in total) were aligned to the SILVA 16S rRNA gene database^6^ (SILVA 132) using the command ‘qiime feature-classifier classify-sklearn’. Samples with total read count below 8.000 were considered to have insufficient coverage and were removed from further analysis.

Three water contaminants were detected across the 38 negative control samples: *Halomonas* (average read count in negative controls 496; in patient samples 21), *Pseudomonas* (average read count in negative controls 132; in patient samples 44), *Shewanella algae* (average read count in negative controls 424; in patient samples 71). *Pseudomonas* and *Halomonas* were not identified to the species level. In order to correct for the contamination, the genera *Halomonas*, *Pseudomonas* and *Shewanella* were removed from all further analyses.

Raw sequencing data will be made available on the European Nucleotide Archive, project code PRJEB34684.

**1.5 Chest CT scores**

HRCT scans were performed for routine diagnostic screening every 5 years. For 74 patients, one scan closest to time of oropharyngeal sampling was scored by a pulmonary radiologist (F.M.H.) for AD and ILD in each lobe using a previously published scoring system ^7,8^. AD was scored as extent and severity of bronchiectasis, airway wall thickening, mucus plugging, tree-in-bud and airtrapping. ILD was scored as extent and severity of opacities, ground glass, septa thickening and lung nodules. The obtained score was normalised by the maximum obtainable score. For one patient who had undergone lobectomy and one with atelectasis of a single lobe, the maximum obtainable score was adapted to exclude the missing lobe. In thirteen cases where expiratory scans were not available, airtrapping could not be evaluated and this element was removed from the score.

## 1.6 Data analysis and statistical methods

## All analyses were performed using R 3.2.0^9^, and made publically available on our group’s Gitlab page: [www.gitlab.com/rberbers/cvid_mbiota_oral](http://www.gitlab.com/rberbers/cvid_mbiota_oral) . Continuous baseline parameters, bacterial load, alpha diversity and AD/ILD scores were compared using the Mann-Whitney rank test or Student’s t-test depending on distribution of the data. Categorical variables were compared using a two-tailed Fisher’s exact test. Alpha diversity was calculating using the inverse Simpson index using the package *vegan*. Principal Component Analysis (PCA) was performed using the *prcomp* function on the centered log ratio (CLR) transformed data^10^. Count zero multiplicative replacement (CZM) from the package *zCompositions* was used to replace zeroes prior to CLR transformation. PERMANOVA was used to detect overall differences in PCA using the *adonis* function in *vegan*. Differential abundance testing was performed using ANCOM^11^ with Benjamini-Hochberg correction for multiple testing using an alpha of 0.05 as a threshold for significance. Variables with percentage zeroes greater than 25% of all samples were excluded from analysis. All ANCOM analyses were corrected for age and gender.

Correlation between lung scores and microbiota were done on the CLR-transformed sequencing data as described above. Linear regression was performed using the function *lm*() and the following model: [lung score] ~ gender + age + [bacterium]

Bootstrapped confidence intervals were generated using the function *boot*() and 1000 iterations. Benjamini Hochberg correction was used to correct for false discovery rate.

**1.7 Supplementary references**

1. Wyllie, A. L. *et al.* Streptococcus pneumoniae in Saliva of Dutch Primary School Children. *PLoS One* **9**, e102045 (2014).

2. Liu, C. M. *et al.* BactQuant : An enhanced broad-coverage bacterial quantitative real-time PCR assay. *BMC Microbiol.* **12**, 56 (2012).

3. Fadrosh, D. W. *et al.* An improved dual-indexing approach for multiplexed 16S rRNA gene sequencing on the Illumina MiSeq platform. *Microbiome* **2**, 1–7 (2014).

4. Bolyen, E. *et al.* QIIME 2 : Reproducible , interactive , scalable , and extensible microbiome data science. *PeerJ Prepr.* (2018).

5. Callahan, B. J. *et al.* DADA2 : High-resolution sample inference from Illumina amplicon data. *Nat. Methods* **13**, 581–587 (2016).

6. Quast, C. *et al.* The SILVA ribosomal RNA gene database project : improved data processing and web-based tools. *Nucleic Acids Res.* **41**, 590–596 (2013).

7. Ven, A. A. J. M. Van De & Montfrans, J. M. Van A CT Scan Score for the Assessment of Lung Disease in Children With Common Variable Immunodeficiency Disorders. *Chest* **138**, 371–379 (2010).

8. Maarschalk-Ellerbroek, L. J. *et al.* CT Screening for Pulmonary Pathology in Common Variable Immunodeficiency Disorders and the Correlation with Clinical and Immunological Parameters. *J. Clin. Immunol.* **34**, 642–654 (2014).

9. R Foundation for Statistical Computing, Vienna, A. R: A language and environment for statistical computing. (2018).at <https://www.r-project.org/>

10. Aitchison, J. *The statistical analysis of compositional data: Monographs on statistics and applied probability*. (Chapman & Hall Ltd., London, 1986).

11. Kaul, A., Mandal, S., Davidov, O. & Peddada, S. D. Analysis of Microbiome Data in the Presence of Excess Zeros. *Front. Microbiol.* **8**, 2114 (2017).

# Supplementary Tables

**Table S1: study characteristics for the sequencing cohort**

|  | **HC** | **CVID +IgA (IgA >0.1 g/L)** | **CVID -IgA (IgA <0.1 g/L)** | **XLA** |
| --- | --- | --- | --- | --- |
| **Total N** | 41 | 48 | 33 | 11 |
| **Age: mean ± SD** | 41 **±** 12 | 34 **±** 19 | 39 **±** 14 | 23 **±** 15 |
|  |  |  |  |  |
| **Male % (N)** | 27% (11/41) | 52% (25/48) | 61% (20/33) | 100% (11/11) |
| **Medication use during 3 months prior to sampling: % (N)** | | | | |
| **Antibiotics** | 0% (0/41) | 33% (16/48) | 24% (8/33) | 55% (6/11) |
| **Immune suppressive therapy** | 0% (0/41) | 13% (6/48) | 15% (5/33) | 8% (1/12) |
| **Clinical phenotype: % (N)** | | | | |
| **Any inflammatory complication** | 0% (0/41) | 31% (15/48) | 67% (22/33) | 9% (1/11) |
| **Autoimmune disease** | 0% (0/41) | 19% (9/48) | 36% (12/33) | 9% (1/11) |
| **GLILD (clinical diagnosis)** | 0% (0/41) | 6% (3/48) | 6% (2/33) | 0% (0/11) |
| **Granulomatous disease other** | 0% (0/41) | 2% (1/48) | 6% (2/33) | 0% (0/11) |
| **Enteritis** | 0% (0/41) | 15% (7/48) | 27% (9/33) | 0% (0/11) |
| **Malignancy** | 0% (0/41) | 6% (3/48) | 3% (1/33) | 0% (0/11) |
| **IgA status:** | | | | |
| **Serum IgA levels available (N)** | 80% (34/41) | 100% (48/48) | 100% (33/33) | 100% 11/11 |
| **% serum IgA low (<0.1 g/L)** | 0% (0/34) | 0% (0/48) | 100% (33/33) | 100% (11/11) |
| **Serum IgA mean ± SD in g/L** | 2.04 **±** 0.79 | 0.75 **±** 0.58 | 0.07 **±** 0.00 | 0.07 **±** 0.00 |

## Supplementary Figures


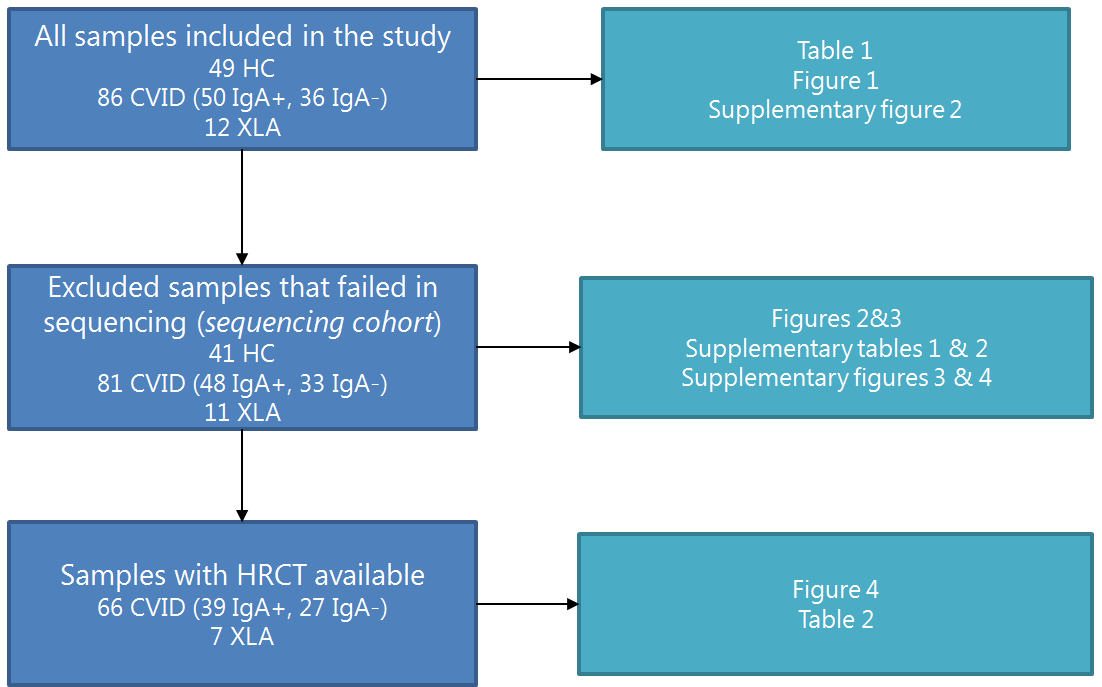


**Supplementary Figure 1.** Overview of all samples used in each figure and table. HC: healthy control. CVID: common variable immunodeficiency. XLA: X-linked agammaglobulinemia. IgA: immunoglobulin A. HRCT: high-resolution computed tomography.


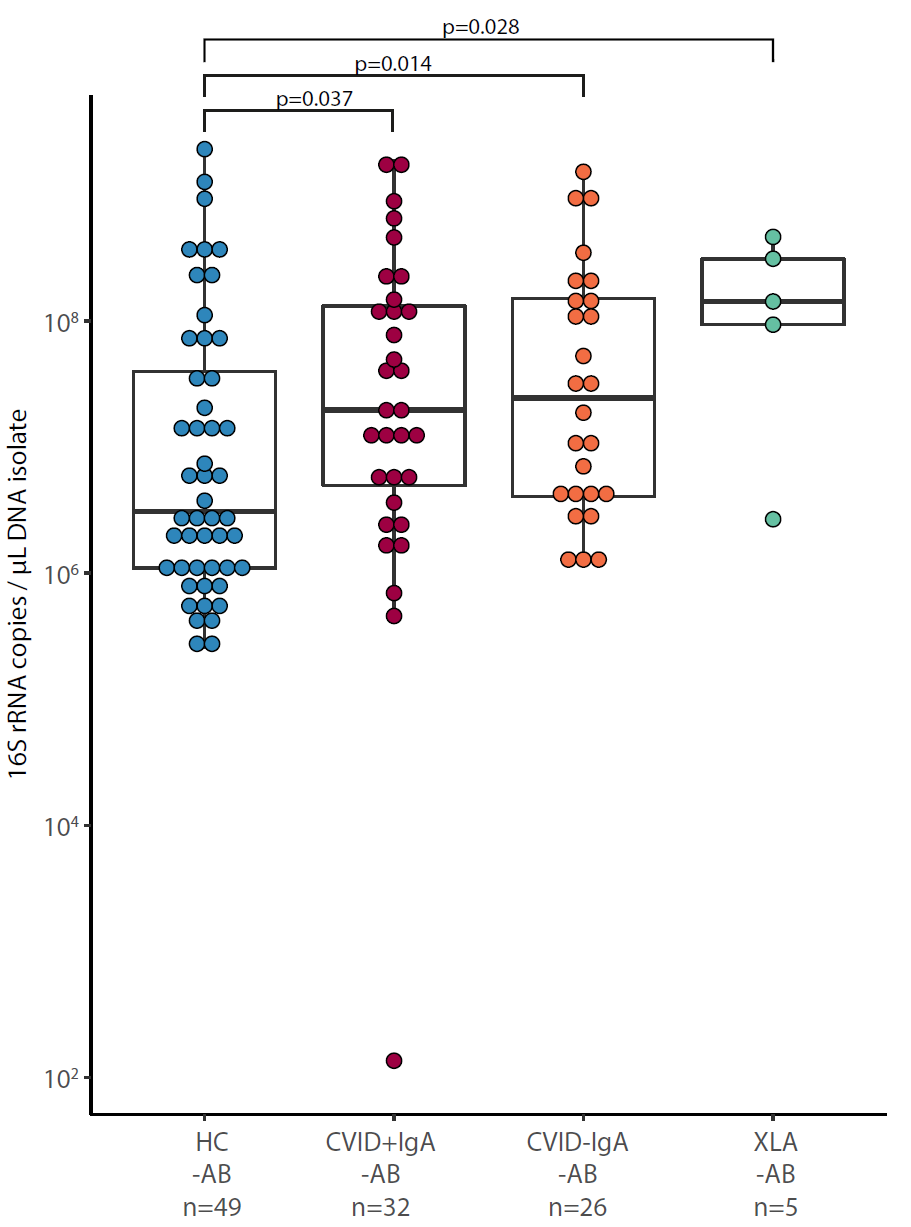


**Supplementary figure 2**: Bacterial load in oropharyngeal swab samples as determined by qPCR for copies of the 16S rRNA gene in DNA isolates from oropharyngeal swabs in participants who did not use antibiotics (-AB) up to three months prior to sampling. Healthy controls (HC, n=49), CVID +IgA (n=32), CVID –IgA (n=26) and X-linked agammaglobulinemia (XLA, n=5). The horizontal line inside the box represents the median. The whiskers represent the lowest and highest values within 1.5×interquartile range. Statistical test: Mann-Whitney U test.


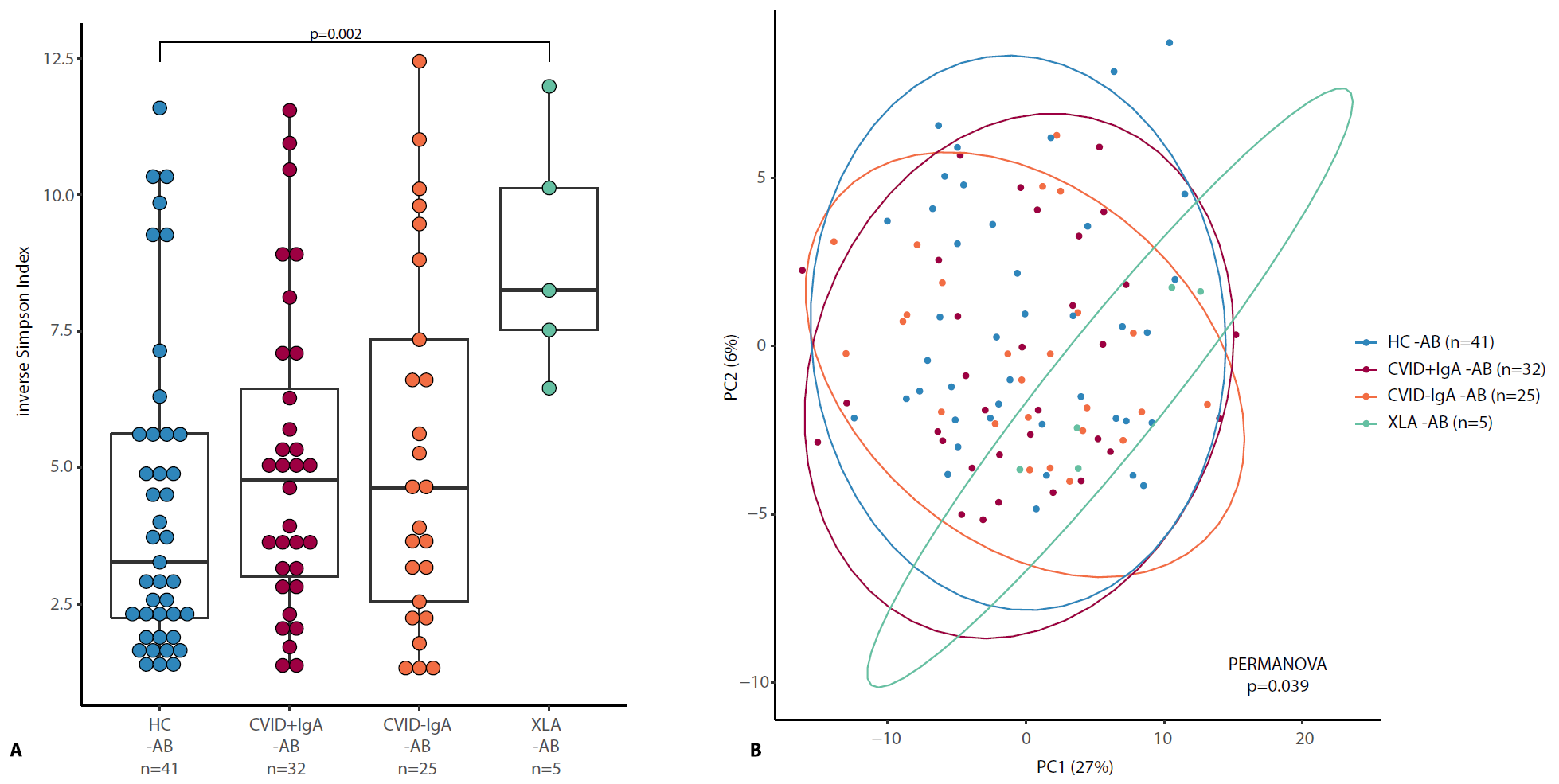


**Supplementary figure 3**

**A** Alpha diversity as measured by inverse Simpson’s index on 16S rRNA sequencing data of participants who did not use antibiotics up to 3 months prior to sampling (-AB): healthy controls (HC, n=41), CVID +IgA (n=32), CVID -IgA (n=25) and XLA (n=5). The horizontal line inside the box represents the median. The whiskers represent the lowest and highest values within 1.5×interquartile range. Statistical test: Mann-Whitney U test.


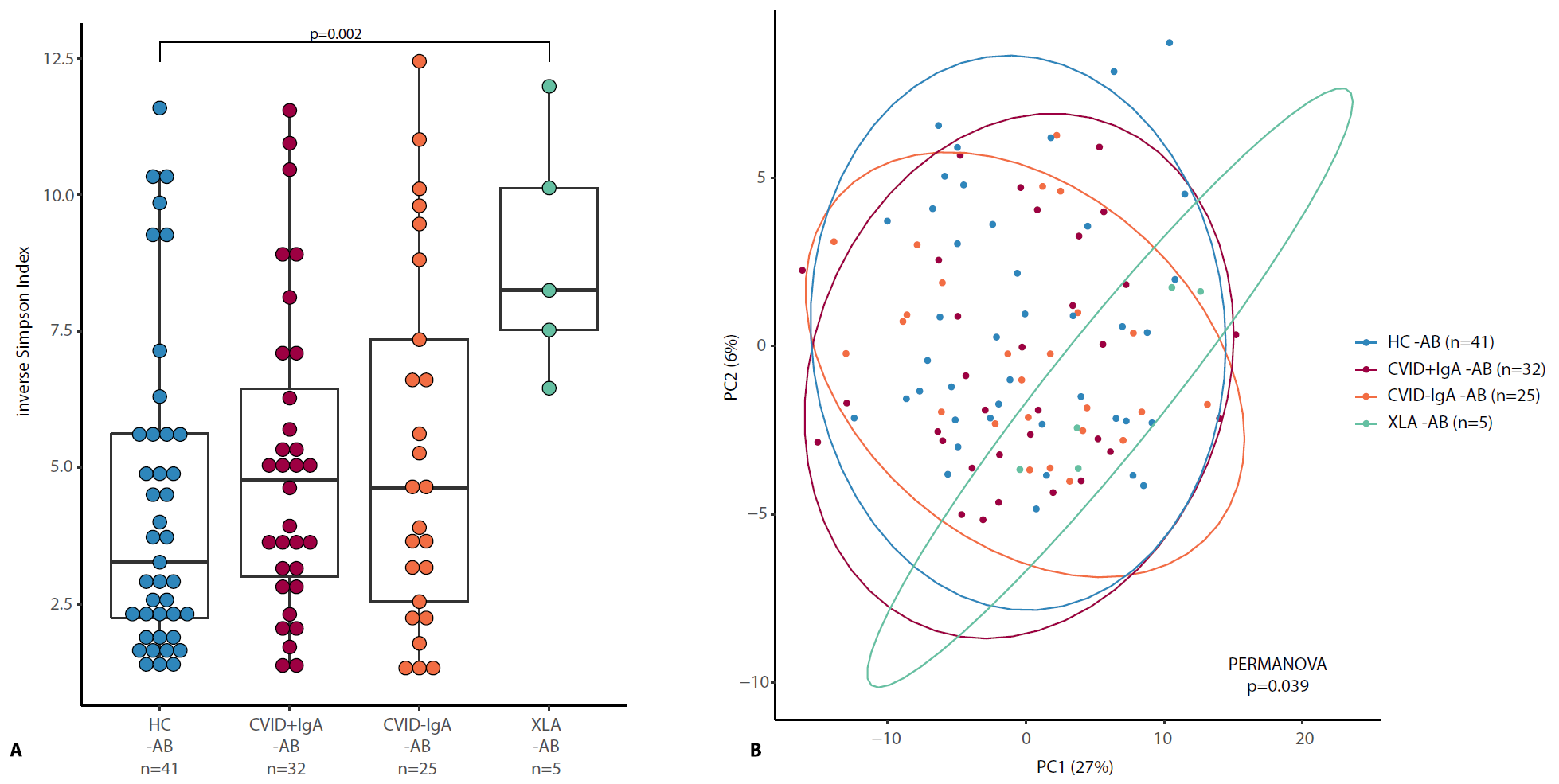


**Supplementary figure 3**

**B** Principal component analysis of centered log ratio (CLR)-transformed family level 16S rRNA sequencing data of the same samples described in **A**. Ellipses indicate 95% confidence intervals. Statistics: PERMANOVA using Euclidean distance on CLR data, p=0.015.


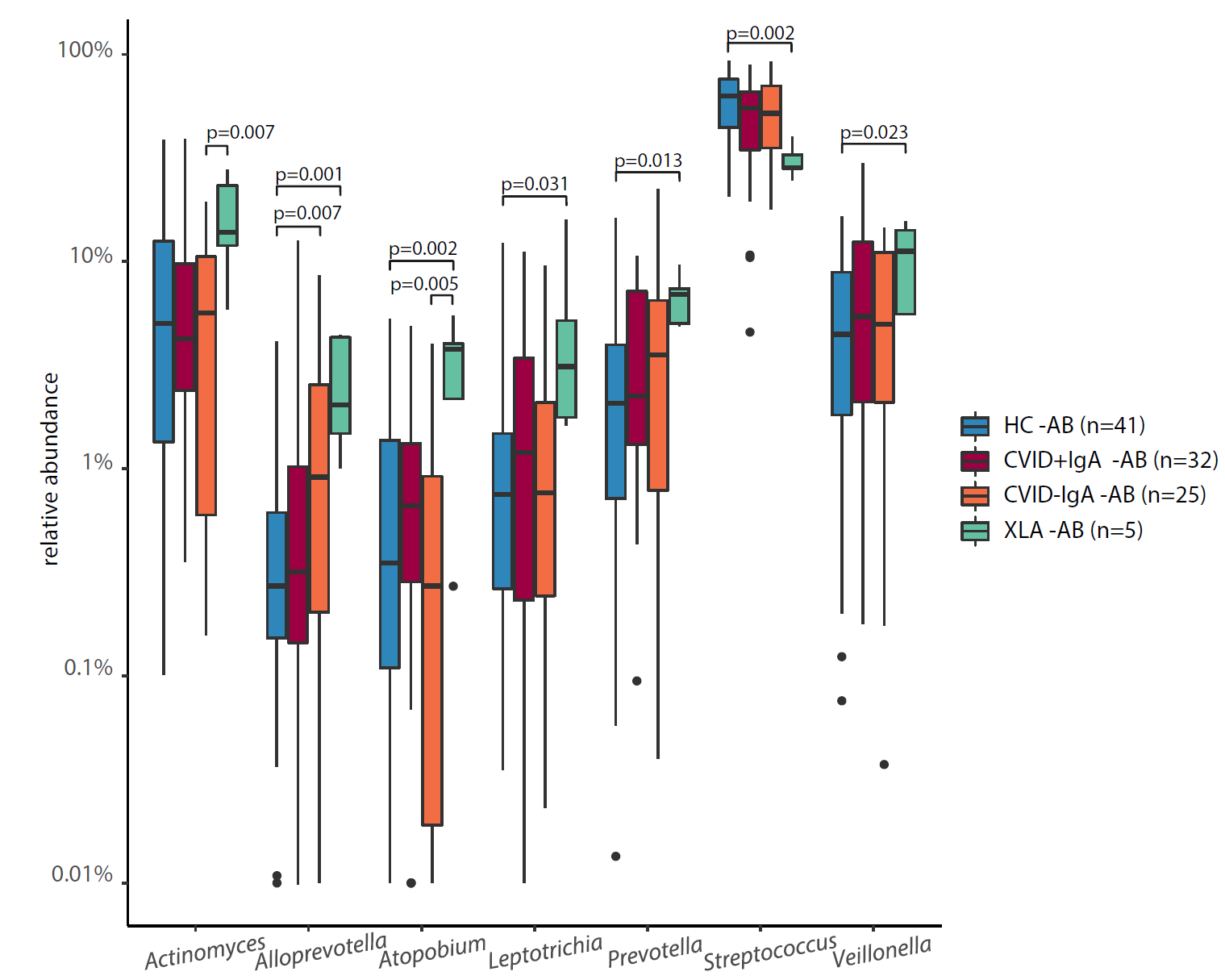


**Supplementary figure 4** Differentially abundant taxa in 16S rRNA sequencing of healthy controls (HC, n=41), CVID +IgA (n=32), CVID -IgA (n=25) and XLA (n=5) patients who did not use antibiotics (-AB) 3 months prior to sampling. Statistics: ANCOM corrected for age and gender and Benjamini-Hochberg correction for false discovery rate. The horizontal line inside the box represents the median. The whiskers represent the lowest and highest values within 1.5×interquartile range.


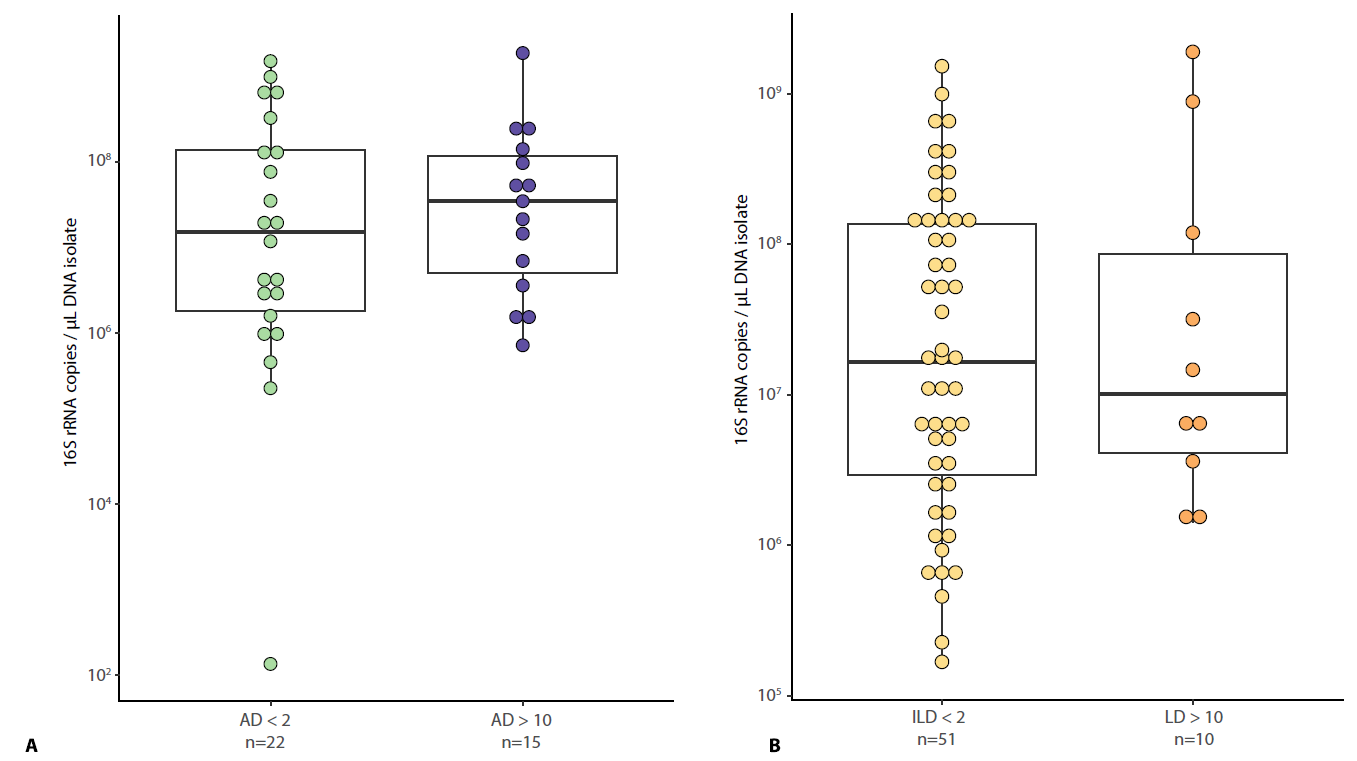


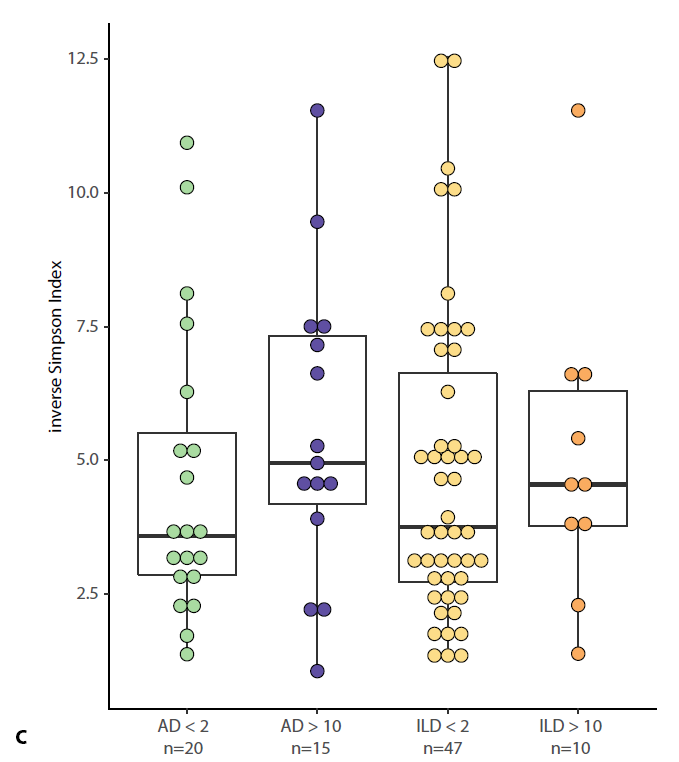
 **Supplementary figure 5 A** Bacterial load in oropharyngeal swab samples as determined by qPCR for copies of the 16S rRNA gene in DNA isolates from oropharyngeal swabs in patients with airway disease score (AD) <2 (n=22) or >10 (n=15).

**B** Bacterial load in oropharyngeal swab samples as determined by qPCR for copies of the 16S rRNA gene in DNA isolates from oropharyngeal swabs in patients with interstitial lung disease score (ILD) <2 (n=51) or >10 (n=10).

**C** Alpha diversity of the same samples described in **A and B** as measured by inverse Simpson’s index on 16S rRNA sequencing data.

The horizontal line inside the box represents the median. The whiskers represent the lowest and highest values within 1.5×interquartile range. Statistical test: Mann-Whitney U test.
